# Hepatitis B Virus Genotype G forms core-like particles with unique structural properties

**DOI:** 10.1111/j.1365-2893.2010.01330.x

**Published:** 2011-06

**Authors:** J J H Cotelesage, C Osiowy, C Lawrence, S L deVarennes, S Teow, D R Beniac, T F Booth

**Affiliations:** 1 Viral Diseases Division, National Microbiology Laboratory, Public Health Agency of CanadaWinnipeg, Manitoba, Canada; 2 Department of Medical Microbiology, University of ManitobaWinnipeg, Manitoba, Canada

**Keywords:** core-like particles, electron microscopy, Hepatitis B virus

## Abstract

**Summary:**

We have determined the structure of the core capsid of an unusual variant of hepatitis B virus, genotype G (HBV/G) at 14 Å resolution, using cryo-electron microscopy. The structure reveals surface features not present in the prototype HBV/A genotype. HBV/G is novel in that it has a unique 36- bp insertion downstream of the core gene start codon. This results in a twelve amino acid insertion at the N-terminal end of the core protein, and two stop codons in the precore region that prevent the expression of HBeAg. HBV/G replication in patients is associated with co-infection with another genotype of HBV, suggesting that HBV/G may have reduced replication efficiency *in vivo*. We localized the N-terminal insertion in HBV/G and show that it forms two additional masses on the core surface adjacent to each of the dimer-spikes and have modelled the structure of the additional residues within this density. We show that the position of the insertion would not interfere with translocation of nucleic acids through the pores to the core interior compartment. However, the insertion may partially obscure several residues on the core surface that are known to play a role in envelopment and secretion of virions, or that could affect structural rearrangements that may trigger envelopment after DNA second-strand synthesis.

## Introduction

Hepatitis B virus (HBV) is one of the most important causes of liver disease in humans. Although HBV infection is preventable by vaccination, it currently affects more than 350 million people worldwide, with many infected as long-term carriers [1]. HBV is a member of the *Hepadnaviridae* with a partially double stranded DNA genome of 3.2 kb in size [2]. The HBV nucleocapsid core is icosahedral and enveloped by a membrane containing the surface antigen glycoprotein (HBsAg). At the structural level, the nucleocapsid of the prototype HBV (genotype A) has been studied extensively by both cryo-electron microscopy (cryo-EM) and X-ray crystallography [2–4]. In addition to the presence of HBsAg, two other diagnostic markers can frequently be detected in the serum of infected patients: antibody directed against the core antigen (HBcAg, consisting of assembled capsids) and the e antigen (HBeAg) an alternate translation product of the core gene that is cleaved and secreted posttranslationally. The HBcAg is dimeric, and assembled cores exhibit oligomeric dimorphism: a proportion has T = 3 symmetry (with 180 subunits, consisting of 90 dimers of HBcAg) and the other proportion having T = 4 symmetry (240 subunits and 120 dimers) [5]. This icosahedral dimorphism is present both in cores that are purified from the serum of HBV-infected patients and in core-like particles which self-assemble upon *in vitro* expression of the core protein. When the HBV virion enters cells, the uncoated core is transported to the nucleus where HBV genomic DNA is repaired to the covalently closed circular DNA form. During replication, the RNA pregenome is incorporated into assembling cores, and reverse transcription to form the genomic DNA occurs within the assembled core [2]. The core protein dimers form spike-like protrusions which extend from the spherical surface of the core particle, and also form a network of pores or holes [2].

The presence of different genotypes of HBV has been correlated with different geographical regions and with varying severity of disease [6]. Recently, a novel genotype of HBV (HBV/G) was described [7], which has a unique 36- bp insertion downstream of the core gene start codon, resulting in a twelve amino acid insertion at the N-terminal end of the core protein. In addition, HBV/G has two stop codons in the precore region at positions 2 and 28, which prevent the expression of HBeAg [8]. The fact that HBV/G is almost exclusively detected in patients in association with one of the other seven HBV genotypes (usually HBV/A), and the frequent presence of HBeAg in HBV/G-infected individuals, suggests that HBV/G replication requires co-infection with a helper virus of another HBV genotype [9,10]. The unique epidemiological and clinical profile of HBV/G suggested that it could be deficient in some other aspects of its function, perhaps as a result of the differences in the capsid structure and function caused by the presence of the N-terminal insertion in the core protein. Although HBV/G has been shown to be capable of replication in the absence of another co-infecting genotype *in vitro*, the envelopment of genotype G appeared to be less efficient than that of genotype A, while the presence of the 36-nt insertion enhanced core protein expression and genome replication [9]. The aim of this study was to investigate for the first time the structural morphology of the HBV/G core particles in comparison with HBV/A, and in particular to localize the 12 amino acid insertion. We used cryo-electron microscopy and structural modelling to investigate structural differences in the features of HBV/G core-like particles that could influence functions such as replication, genome packaging, or possibly HBsAg envelopment of the capsid and viral secretion.

## Materials and methods

### Preparation and purification of core protein from HBV Genotype G

HBV/G-infected individuals were identified through routine diagnostic testing and strain surveillance at the National Microbiology Laboratory (NML) using sequencing and phylogenetic analysis [10]. The gene coding for the HBV/G core protein was PCR amplified, cloned into the pET28b vector (Novagen, EMD Biosciences, Gibbstown, NJ, USA) and expressed in *Escherichia coli* strain BL21 (DE3). Clarified culture lysates were centrifuged on a 20–60% sucrose gradient at 30 000 rpm in a Beckman SW4OTi rotor for 14 h. Fractions were analysed by SDS–PAGE and Western blotting with anti-HBc antibody (MA1-21697; Pierce Antibody, Rockford, IL, USA). Cores were dialysed against PBS pH 7.4 using a 10 K MW cut off Pierce Slide-A-Lyzer cassette (Thermo Scientific, Waltham, Massachusetts, USA). The HBV/G core sequence was submitted to the National Center for Biotechnology Information GenBank database under accession number GU325783.

### Cryo-EM sample preparation and data collection

**Fig. 1:**
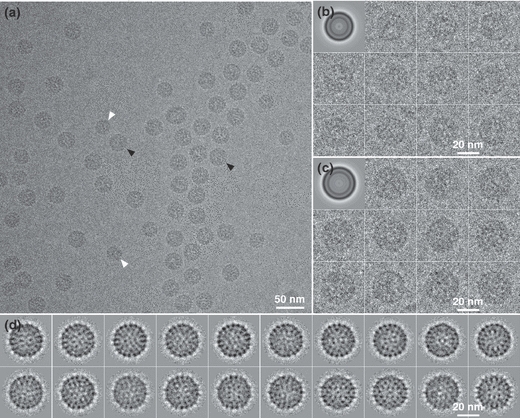
(a) Cryo-electron microscopy of HBV/G core-like particles. T = 3 particles (white arrows) and T = 4 particles (black arrows) are shown. Panels (b) and (c) illustrate the sorting of the T = 3 capsids from T = 4 capsids, respectively. The rotational average (top left in panels b and c) was used as a reference for sorting. Several representative sorted particles are presented. Characteristic class averages generated by projection matching for the T = 4 reconstruction are shown in (d).

Cryo-EM and specimen preparation was performed as previously described [11,12]. Briefly, specimens on glow-discharged Quantifoil grids (Quantifoil MicroTools GmbH, Jena, Germany) were plunge cooled in liquid ethane using a Vitribot Mark IV (FEI Company). Specimens were imaged at 200 kV in a FEI Tecnai 20G^2^ electron microscope equipped with a 4K CCD camera (FEI Company, Hillsboro, Oregon, USA). Images were recorded at a magnification of 80 000×, (corresponding to 108 240× at the CCD detector, or 1.353 Å/pixel) using a total dose of 10 electrons/Å^2^ and a defocus of 3.5–6 μ. A total of 13 455 particles were analysed. Correction was made for the contrast transfer function using EMAN [13]. T = 3 or T = 4 capsids were separated in SPIDER [14] (Fig. 1b,c). Two populations of 1887, and 11 568 images of T = 3 and T = 4 capsids, respectively, were analysed.

### Image processing

The T = 4 capsids were analysed, and class averages were calculated for the twofold, threefold and fivefold axes of symmetry. A preliminary icosahedral 3D model was visually inspected using the Chimera software package [15]. Data were then analysed using projection matching [14,16]. Between each refinement cycle the software package SITUS [17] was used in parallel to select the contiguous mass which corresponded to a single T = 4 HBV/G capsid.

### Resolution, and comparison of the HBV/A and HBV/G models

Resolution of the structure was estimated by plotting the Fourier shell correlation (FSC) against resolution, giving a value of 14 Å at an FSC of 0.5. The resolution of the HBV/A model 1QGT.pdb determined by X-ray crystallography [4] was then reduced to 14 Å using the EMAN program ‘pdb2mrc’ [13]. Both maps were normalized for electron density, aligned and compared by creating a difference map. The contour level of the difference map was selected so that its mass matched the volume of the additional 12 residue N-terminal insert of the HBV/G core structure [17]. The tertiary structure of the 12 residues was predicted by the I-TASSER server [18], and the resulting coordinates were modelled in to the difference map density HBV/G structure using the program COOT [19].

## Results

Cryo-EM revealed that the HBV/G core protein was able to form T = 3 and T = 4 capsid structures similar to those formed by genotype A (Fig. 1). Thus, we confirmed that the additional 12 N-terminal residues do not impair capsid formation. Furthermore, the insertion does not affect the basic structure of the core, other than presenting two additional regions of mass at the base of each of the spikes. Several groups have previously engineered N-terminal extensions to the HBcAg and found that this did not impair self-assembly of the expressed proteins into core-like particles [20,21]. The majority of the HBV/G cores had T = 4 morphology, as previously reported in expressed core–like particles, and in cores isolated from patient specimens [22].

**Fig. 2:**
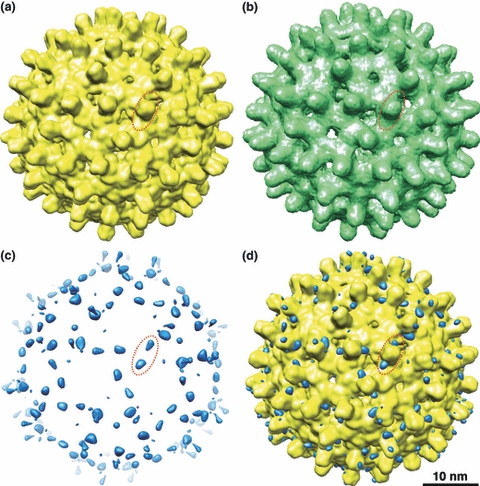
Surface shaded representation of the HBV/A capsid structure solved by X-ray crystallography (yellow), and HBV/G capsid structure solved by cryoEM (green) are shown in (a) and (b), respectively. Structures are Fourier filtered to 14 Å resolution for comparison. A difference map (c, shown in blue) was created by subtracting the HBV/A capsid structure from the HBV/G core structure is presented with a mass threshold set to account for the 12 extra residues from the HBV/G cryo-EM map. Panel (d) shows the difference map (blue) superimposed over the HBV/A core crystal structure (yellow). In all four panels, a red oval has been superposed over the reconstruction to highlight a single dimer spike in the structures at the base of the spike.

**Fig. 3:**
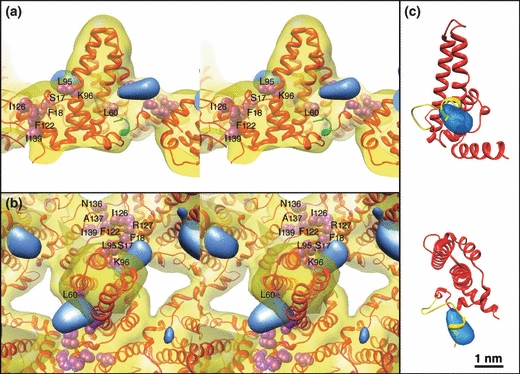
(a) and (b). Stereo images of the HBV/A core protein structure. The red ribbon is the HBV/A core crystal structure, PDB accession 1QGT, and the yellow density is the structure derived from the HBV/A core X-ray crystallography data displayed at 14 Å resolution, to match resolution of the difference map shown in blue. The additional mass found on HBV/G (blue density) was determined by subtracting the HBV/A structure from the HBV/G structure. (a) The mass from the extra 12 amino acids on the N-terminal end of HBV/G is near to the N-terminal end of the HBV/A core (the portion of the ribbon coloured green). (b) Top view looking down one of the spike 2-fold axes. Locations of the amino acids critical for the encapsidation of the mature HBV particle, and that interact with the cytoplasmic domains of the HBsAg, are shown as purple spheres. The extra mass from the 12 amino acid insert may stearically interfere with access to some of these residues (notably S17, F18, L60, L95). (c) Side and top views of HBV/G core protein model (red ribbon) with the12 residue N-terminal insertion (yellow ribbon) modelled into the difference map density (transparent blue). Only one copy of the core molecule within the dimer is shown for clarity.

Superficially, the three-dimensional structure of the T = 4 capsid formed by HBV/G core protein looks very similar to the crystal structure of the HBV/A capsid, PDB accession 1QGT (Figs 2a,b) [4]. The difference map created shows additional mass around the base of the spikes of the HBV core protomers (Figs 2c,d). When the difference map is superimposed with the HBV/A atomic coordinates the additional mass of the HBV/G structure is found to be adjacent to the N-terminal residues of the core protein (Fig. 2d and Figs 3a, b). The extra mass protrudes from the surface of the core particle and sits beside the capsid pores and not over them, so this feature would not effectively reduce the diffusive movement of nucleotide triphosphates into the core interior, or affect the release of nucleic acids from the core (Fig. 3a, b). When modelling the extra 12 residues by TASSER/COOT, it was apparent that there are a number of possible arrangements that 12 residues can take up and still be contained within the corresponding density (Fig. 3c). Previously, a crystal structure of an HBV capsid protein engineered with an additional 11 residue extension to its N-terminal end has been determined, PDB accession 2QIJ [21]. Although the orientation was different from that observed in HBV/G, both additional masses were pointing away from the four-helix bundle as opposed to interacting with the main body of the core protein (Fig. 3c).

## Discussion

The possible function or role that the additional N-terminal mass in the core protein of HBV/G may play in replication, and the reason for its retention only in the HBV/G strain is unclear. However, the 36-nt core insertion on HBV/G appears to result in less efficient envelopment of mature virions compared to that of HBV/A [9].

The HBV capsid plays an important role in envelopment of the virion with membrane containing the HBsAg, which is a prerequisite for the secretion of viable progeny virions [2]. Amino acids in the core protein that are involved in envelopment competence, and which presumably make important contacts with the cytoplasmic domain of membrane-bound preS1 protein, have been mapped, both by mutagenesis [23–25] and by analysis of naturally occurring patient-derived HBV mutants that vary in their capacities to secrete mature virions [26–28]. There are thus 11 residues that have been shown to be essential for envelopment but were nonessential for capsid formation. A number of these 11 residues (shown in purple in Figs 3a, b) are found near the electron density made by the extra N-terminal insertion peptide of HBV/G (Fig. 3b). It is tempting to speculate that the 12 amino acid N-terminal insertion in HBV/G could be responsible for partially reducing the efficient envelopment of HBV/G, because of its proximity to several of these key residues that make contacts with the cytoplasmic domain of HBsAg. In particular, the N-terminal insertion partially covers several of these residues that were shown to be involved in secretion of mature particles, notably S17, F18, L60 and L95 (Fig. 3b) and thus might sterically hinder interactions between the L protein and core particle of HBV/G during envelopment. In addition, in previously published work, Hui *et al.* [20] showed that an insertion of 23 amino acids engineered on the N-terminal end of the HBV core protein could prevent core envelopment by membranes containing HBsAg. The additional N-terminal residues in HBV/G could interfere with one or more of the essential contacts that are important for core envelopment, leading to attenuated or partially deficient virion maturation. Our data show that the N-terminal insertion in the core of HBV/G partially covers the ‘hydrophobic pocket’ which contains the residues that are essential for normal virion secretion [22]. This pocket has been shown to undergo structural changes after envelopment, which may act as a trigger, to ensure that envelopment does not take place until after DNA second-strand synthesis has completed [22]. In HBV/G the N-terminal insertion might therefore also interfere with these structural changes, as well as sterically hindering key amino acids that are thought to interact with the envelope. A reduced ability for virion envelopment and secretion might partially explain why the presence of HBV/G in patients is strongly associated with co-infection by another genotype.

Our structural analysis shows that specific antibodies or other ligands directed against the 12 amino acid insertion would be able to readily bind without stearic hindrance. This unique peptide that is exposed on the surface of the HBV/G core could be exploited as a possible site for epitope presentation in an HBcAg core–based vaccine. Because HBV/G already has an insertion in the N-terminus, it may tolerate even larger insertions than have been tried previously in HBV/A cores, while still maintaining the ability for efficient self-assembly. In addition, the possibility of detecting specific antibodies directed against the insertion peptide in sera from patients infected with HBV/G could be explored. Such testing might help to resolve the issue of whether HBV/G can replicate *in vivo* in the absence of a helper virus strain and would assist in diagnosing patients infected with HBV/G.

## Abbreviations

FSC: Fourier shell correlation
HBV: Hepatitis B virus
HBcAg: consisting of assembled capsids
HBeAg: e antigen
HBsAg: surface antigen glycoprotein
